# NF2 blocks Snail-mediated p53 suppression in mesothelioma

**DOI:** 10.18632/oncotarget.3543

**Published:** 2015-03-12

**Authors:** Jung-Hyun Cho, Su-Jin Lee, Ah-Young Oh, Min-Ho Yoon, Tae-Geun Woo, Bum-Joon Park

**Affiliations:** ^1^ Department of Molecular Biology, Graduated School of System Biology, College of Natural Science, Pusan National University, Busan

**Keywords:** mesothelioma, p53, NF2, RKIP, Snail

## Abstract

Although asbestos causes malignant pleural mesothelioma (MPM), rising from lung mesothelium, the molecular mechanism has not been suggested until now. Extremely low mutation rate in classical tumor suppressor genes (such as p53 and pRb) and oncogenes (including Ras or myc) indicates that there would be MPM-specific carcinogenesis pathway. To address this, we treated silica to mimic mesothelioma carcinogenesis in mesothelioma and non-small cell lung cancer cell lines (NSCLC). Treatment of silica induced p-Erk and Snail through RKIP reduction. In addition, p53 and E-cadherin were decreased by silica-treatment. Elimination of Snail restored p53 expression. We found that NF2 (frequently deleted in MPM) inhibited Snail-mediated p53 suppression and was stabilized by RKIP. Importantly, GN25, an inhibitor of p53-Snail interaction, induced p53 and apoptosis. These results indicate that MPM can be induced by reduction of RKIP/NF2, which suppresses p53 through Snail. Thus, the p53-Snail binding inhibitor such as GN25 is a drug candidate for MPM.

## INTRODUCTION

Malignant Pleural Mesothelioma (MPM) is asbestos-induced advanced lung cancer with very poor survival rate [1, 2]. Despite tight-regulation against asbestos, due to wide-used asbestos as insulator in constructions, incidence of MPM will be sustained for considerable period [3-5]. In addition, other materials such as silica and nano-carbon tube are considered to be potential tread for MPM [6, 7]. However, molecular carcinogenic mechanism about asbestos or similar micro-materials-induced mesothelioma has not been clearly demonstrated until now. From the global genetic analysis, Neurofibromatosis 2 (NF2/merlin) and BRCA1 associated protein 1 (BAP1) are represented as frequently altered genes in MPM [8-10]. In addition, chronic inflammation by trapped asbestos or silica is suggested as risk factor [11, 12]. But, in aspect of human carcinogenesis, there is no obvious oncogenic mutation that should provide driving force for tumorigenesis and loss of function in tumor suppressor genes in particular p53 pathway [13]. In fact, it is generally accepted concept that, to progress to malignant cancer, p53 pathway should be inactivated. However, MPM shows extremely low genetic mutation rate in p53 and related signaling components such as MDM2 or p14/ARF [8, 14]. This fact indicates that asbestos or silica may inactivate p53 and activate oncogenes through unusual strategy.

To investigate how MPM gains malignant features without classical genetic mutations, we checked the effect of silica on tumorigenic progression. Although asbestos is more suitable agent for this study, tight restriction for usage of asbestos, we were not able to use asbestos. From the analysis of silica effect, we found that silica itself could reduce p53 expression and Raf kinase inhibitory protein, RKIP. In addition, reduced RKIP was closely related with p53 reduction by Snail and NF2 stabilization. Finally, we revealed that inhibition of Snail-p53 interaction could restore the tumor suppressive role of p53 in MPM cell line. These results would provide new insight for understanding of MPM carcinogenesis as well as new strategy for treatment of MPM.

## RESULTS

### Induction of p-Erk in response to silica

**Figure 1:**
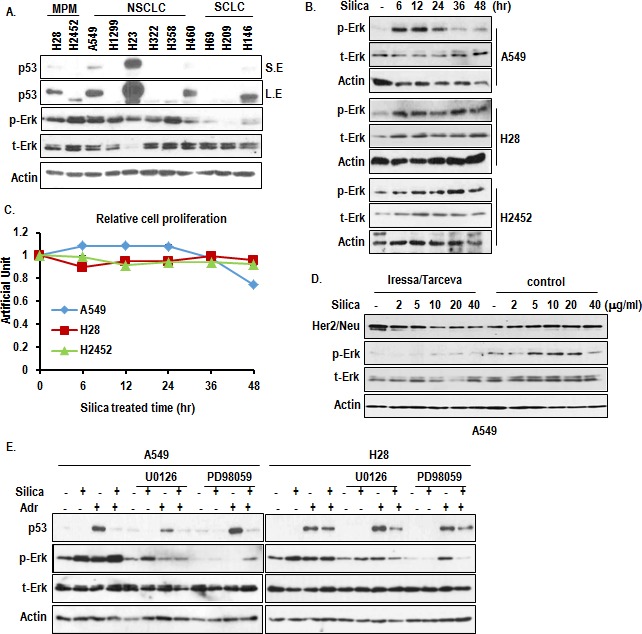
Induction of p-Erk by silica (A) p-Erk is elevated in MPM cell lines as strong as K-Ras mutated cell lines. A549 and H460 are K-Ras mutated cell lines. SCLC cell lines (H69, H209, H146) were used for negative control, because of absence of Ras-related mutation. Indicated protein levels were analyzed by WB. Erk activation was determined by anti-p-Erk specific Ab. t-Erk indicated total Erk. Actin was used for loading control. (B) Silica induces p-Erk in A549. Activation of Erk was monitored by WB analysis with p-Erk after treatment for indicated time in A549 and MPM cell lines. (C) Silica promotes A549 growth at early phase and did not alter the MPM cell line's growth. Cells were seeded at 5×10^5^ cell/well and incubated with 10 μg/ml silica for indicated time. Cell count was performed by tryphan blue dye exclusive using hemocytometer by two independent researchers. (D) Inhibition of RTK using Iressa/tarceva combination treatment did not block the p-Erk increase. RTK inhibitor (10 μM) was treated for 24hr and silica was treated after 1 hr. Actin was used as loading control. (E) MAPK inhibitors cannot suppress silica-induced p-Erk, and silica suppress DNA damage-induced p53. H28 showed the induction of p53 in response to Adr, indicating that this cell line possesses wild type p53. PD98059 (5 μg/ml), U0126 (2 μM), silica and Adriamycin (2 μg/ml) were treated for 24 hr. WB analysis was performed, and Actin was used as loading control.

To obtain the basic information about MPM, we measured the expression of p53 and Erk activation. In two kinds of MPM cell lines, p53 showed quite different expression pattern (Figure 1A). H28 showed very low p53 expression and H2452 expressed small sized p53, despite genetically wild type (Figure 1A) [15, 16]. However, p-Erk expression in MPM was elevated as strongly as K-ras mutated cell lines (A549 and H460), in spite of wild type Ras [17, 18]. Thus, we checked the effect of silica on expression of p-Erk in A549 and MPM cell lines. Treatment of silica on serum free condition could induce p-Erk at early phase and reduced at late phase in A549. However, p-Erk in MPM was not reduced even in late phase (Figure 1B). Consistently with p-Erk, cell proliferation in A549 was induced from 6 hr and declined from 24 hr (Figure 1C and Supplementary Figure 1A). However, silica did not alter the cell proliferation in MPM cell lines (Figure 1C and Supplementary Figure 1A). These results indicate that there is defect in regulation of Erk activity in MPM. So, we first tested the involvement of receptor tyrosine kinase (RTK) activation through the combination treatment of RTK inhibitors (Iressa and tarceva). However, although they could suppress basal p-Erk, silica-induced p-Erk was not blocked by them (Figure 1D). Next, we checked the activation of Erk under MEK1/2 inhibitors treated condition. Despite obvious reduction of basal p-Erk in both cell lines, p-Erk was induced in A549 by silica-treatment under MEK1/2 inhibitor-treated condition (Figure 1E). This result indicates that silica can regulate downstream factor of MEK1/2. Thus, PD98059 or U0126 did not show obvious growth suppression effect on MPM cell lines (Supplementary Figure 1B and 1C). Moreover, p53 was increased by treatment of Adriamycin in H28, similarly to A549 (Figure 1E), implying that H28 possesses wild type p53.

### RKIP is responsible for p-Erk induction by silica

**Figure 2:**
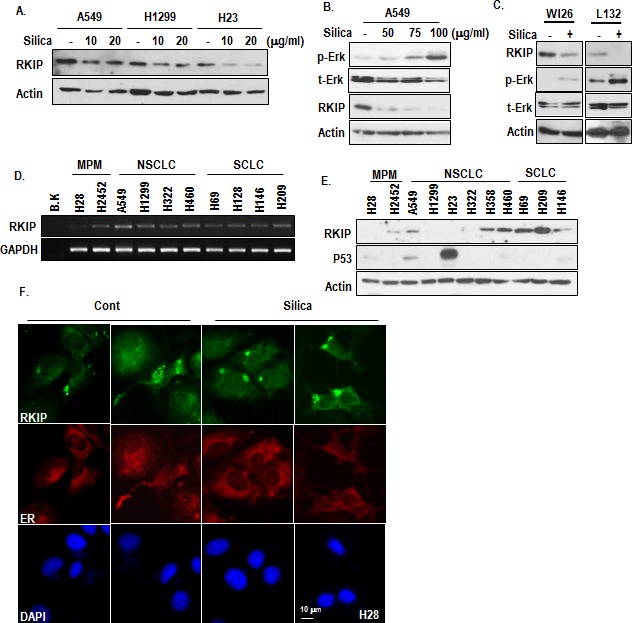
Reduction of RKIP by silica treatment (A) Silica can reduce RKIP expression in 3 kinds of human lung cancer cell lines. Silica was treated for 24 hr in SF condition. The expression of endogenous RKIP was determined by an RKIP specific Ab. (B) RKIP is reduced by silica as dosage-dependent manner. Silica was treated for 24 hr in SF condition. Reversely with RKIP reduction, p-Erk induction was detected as dosage dependent manner. (C) The reduction of RKIP by silica is general event. Non-cancerous human lung epithelial cell lines (WI26 and L132) were incubated with silica (24 hr). WB analysis was performed with indicated antibodies. Similarly with previous result, mutual exclusive expression of RKIP and p-Erk was observed in both cell lines. Actin was used as loading control. (D) Compared to several lung cancer cell line, RKIP transcript is very low in H28 cell line. RT-PCR was performed with matched specific primers. GAPDH was used as loading control. (E) Translational level of RKIP is also low in MPM cell lines. RKIP expression level was monitored by WB analysis. Actin was used as loading control. (F) RKIP forms small spots in cytoplasm of H28. H28 cells were stained with RKIP (green), DAPI (blue) and ER (red).

Since p-Erk is regulated by downstream factor of MEK1/2, we assumed the involvement of RKIP on silica-induced Erk activation and MPM progression. Although it has been reported as inhibitor of Raf-1 [19, 20], RKIP can also inhibit MEK1/2 via direct interaction [21]. To test this, we measured the expression of RKIP in silica-treated NSCLC cell lines and observed the reduction of RKIP (Figure 2A) as dosage dependent manner (Figure 2B). To extend this, we also measured the expression of RKIP in non-transformed lung cells and obtained the same result (Figure 2C). Even in non-lung cancer cell lines such as HCT116 (colon) and AGS (Stomach), RKIP was reduced by silica-treatment (Supplementary Figure S2A). To know how silica suppress RKIP expression, we checked the protein turnover and transcriptional regulation. Proteasome inhibitor did not block the RKIP reduction (Supplementary Figure S2B), whereas RKIP transcript was obviously decreased by silica (Supplementary Figure S2C). So, we next examined the expression of RKIP in MPM, NSCLC and SCLC cell lines and found that similarly with silica-treated cells, RKIP transcript as well as protein level were very low in H28 (Figure 2D and 2E). In addition, several NSCLC cell lines showed the low RKIP expression at protein level (Figure 2E). In immunostaining analysis, we found that RKIP in MPM was located in cytosol as small spots (Figure 2F and Supplementary Figure S2D). Considering other's reports that RKIP is diffused in cytoplasm [22], this feature would be reflected the non-functional portion of RKIP. Indeed, blocking of lysosomal degradation by Bafilomycin A could induce cytoplasmic diffused RKIP as well as protein expression (Supplementary Figure S2E and F). However, autophagy inhibitors (3-MA or rapamycin) did not suppress RKIP spotting (Supplementary Figure S2E and F), suggested that in H28, RKIP was continuously eliminated by lysosomal degradation.

### RKIP regulates p53

**Figure 3:**
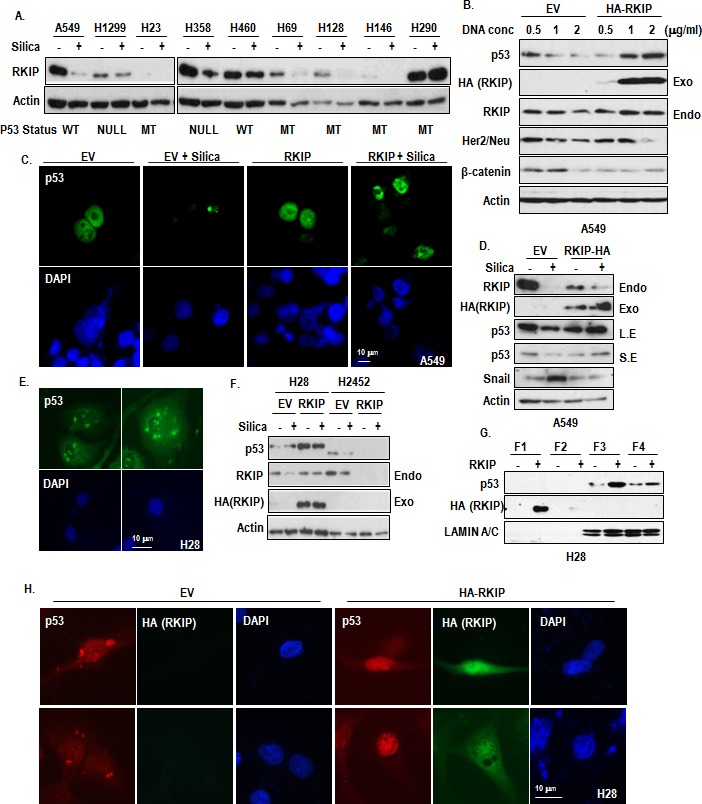
Induction of p53 by RKIP (A) The silica-induced RKIP reduction is not related with genetic status of p53. RKIP reduction was observed in p53 mutant or null cell lines. Reduction of RKIP was monitored by WB analysis after treatment for 24 hr in several lung cancer cell lines. Actin was used for a loading control. p53 status was represented: WT; wild type, MT; mutant, Null; homozygote deletion. (B) RKIP increases p53 expression in a dose-dependent manner. A549 cells were transfected with RKIP as an indicated dose for 24 hr. Actin was used for a loading control. (C) RKIP overexpression blocks silica-induced p53 reduction and retains nuclear p53 localization. HA-RKIP were transfected for 24 hr in A549 cells, and then silica was treated for 24 hr in SF condition. The cells were stained with anti-p53 (DO-1; green), DAPI (blue). (D) Overexpression of RKIP blocks the silica-induced p53 reduction. HA-RKIP were transfected for 24 hr in A549 cells, and then silica was treated for 24 hr in SF condition. WB analysis was performed with indicated antibodies. EV indicates the empty vector and Actin was used as loading control. (E) p53 is located in cytoplasm as small spot or vesicle in H28. The cells were stained with anti-p53 (DO-1; green), DAPI (blue). (F) Overexpressed RKIP induces p53 expression in H28. HA-RKIP were transfected for 24 hr in MPM cells, and then silica was treated for 24 hr in SF condition. Exo and Endo indicated Exogenous and endogenous RKIP, respectively. WB analysis was performed with indicated antibodies. EV indicates the empty vector, and Actin was used as loading control. (G) Obvious increase of nuclear p53 by RKIP transfection in H28. H28 cells were transfected with HA-RKIP for 24 hr. The cells were harvested to isolate the cytoplasm, membrane/organelle, nucleus and insoluble fractions and these samples were analyzed by WB. Each fractions were represented: F1; cytoplasm fraction, F2; membrane/organelle fraction, F3; nucleus fraction, F4; insoluble fraction. (H) RKIP transfection restores the nuclear localization of p53 in H28. HA-RKIP were transfected for 24 hr in H28. The cells were stained with anti-p53 (DO-1; red), anti-HA (green), DAPI (blue).

In previous, we showed that RKIP is a transcriptional target of p53 [23]. Thus, we speculated that reduction of p53 would be one of reason for RKIP reduction. To test this, we first examined the relevance between p53 status and RKIP reduction by silica. In fact, silica could suppress p53 and RKIP expression in A549 (Supplementary Figure S3A) despite prevention of proteasome degradation (Supplementary Figure S3B), suggesting that reduction of p53 by silica might be achieved by MDM2-independent mechanism. However, RKIP reduction by silica did not show any relationship with p53 status (Figure 3A). For example, RKIP was reduced in p53 null H1299 and mutated H23. Instead, transfection of RKIP could increase p53 expression as dosage-dependent manner (Figure 3B). Through IF staining with p53 Ab, we observed that p53 was localized in cytoplasmic droplet when silica was treated (Supplementary Figure S3C). This feature is very similar with Snail-mediated p53 suppression [24]. Thus, we modified our hypothesis that RKIP would be upstream of p53 and tested that RKIP could block the silica-mediated p53 suppression. Ectopic expression of RKIP could be retained in nucleus (Figure 3C) and block the p53 reduction (Figure 3D).

So we next checked the localization of p53 in H28 and H2452. Consistently with silica-treated cells, H28 showed the cytoplasmic spotted p53 (Figure 3E), whereas H2452 showed the cytoplasmic diffused p53 (Supplementary Figure S3D). Similarly with silica-treated cells, ectopic RKIP expression could induce p53 expression in H28 but not H2452 (Figure 3F). Considering these results, H2452 would be developed by different mechanism from RKIP inhibition. In fact, H28 and H2452 are different pathological types (sarcomatoid vs epithelial MPM). Thus, we focused on H28 and re-transfected RKIP into H28. Overexpressed RKIP was located in cytoplasm (Figure 3G and 3H) and induced p53 expression in nucleus (Figure 3G and 3H).

### Snail is responsible for p53 reduction in H28

**Figure 4:**
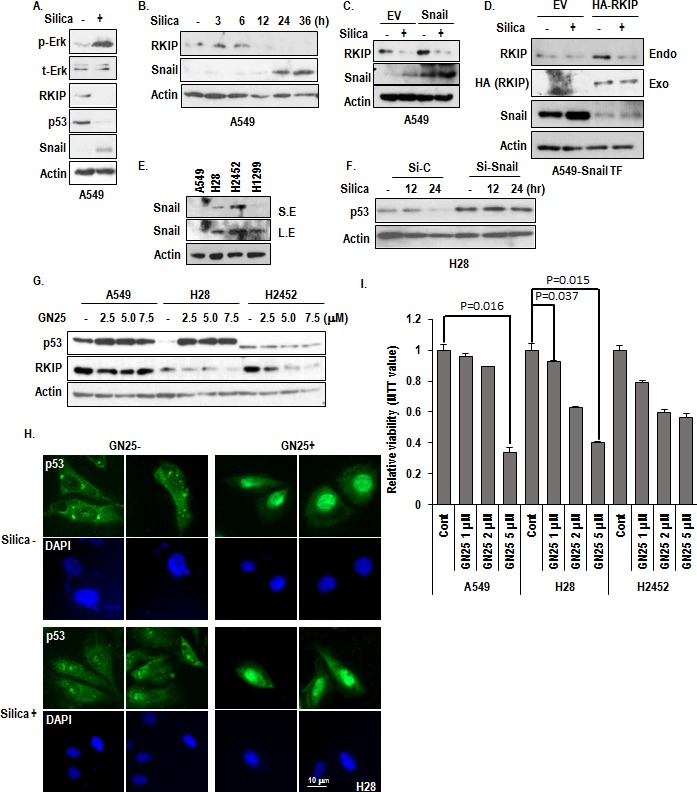
Involvement of Snail in p53 suppression (A) Snail is induced by silica-treatment. Silica was treated for 24 hr in SF condition. The expression of endogenous snail was determined by anti-snail specific Ab. (B) Mutual exclusive expression pattern between RKIP and Snail. The expression of RKIP and Snail were monitored by WB analysis after treatment for indicated time in A549. (C) Snail overexpression cannot alter the RKIP expression. Flag-Snail was transfected for 24 hr in A549 cells. Silica treated for 24 hr in SF condition, and WB was performed. EV indicates the empty vector. Actin was used as loading control. (D) RKIP suppressed Snail expression. HA-RKIP was co-transfected in Snail-transfected A549 cells for 24 hr, and then silica was treated for 24 hr in SF condition, and WB was performed. (E) Snail is elevated in MPM cell lines. Snail expression level was monitored by WB analysis. (F) Snail knock down induces p53 expression in H28. Si-Snail was transfected for 24 hr in H28 cells, and then silica was treated for 12 to 24 hr in SF condition. WB analysis was performed with indicated antibodies, and Actin was used as loading control. (G) GN25 can increase p53 in A549 and H28. After treatment of GN25 (2-7.5 μM) for 12 hr, cells were subjected into WB analysis with indicated antibodies. Actin was used as loading control. (H) p53 expression is increased in nucleus by GN25 treatment, in regardless of silica-treatment. H28 was treated with GN25 (2.5 μM) before an hr to be treated silica. Silica was treated for 24 hr. The cells were stained with anti-p53 (DO-1; green), DAPI (blue). (I) GN25 suppresses H28 viability. After incubation with GN25 (2.5 μM) for 24 hr, the cell viability was monitored by MTT assay.

Since silica promoted the formation of cytoplasmic small vesicular p53 (Figure 3C) and H28 also showed the similar feature (Figure 3E), we assumed that the Snail would be involved in p53 suppression mechanism. In fact, we have revealed that Snail can eliminate p53 by direct interaction and exocytosis [24, 25]. We also observed that the reduction of p53 was not blocked by MG132 (Supplementary Figure 2B), also supporting our hypothesis [25]. As we expected, treatment of silica could induce Snail (Figure 4A) and Snail expression showed the mutual exclusive pattern with RKIP following incubation time (Figure 4B). In addition, we observed the increase of exogenously transfected Snail by silica (Figure 4C), indicating that Snail would be regulated at post-transcription level. Moreover, Snail transfection did not alter the RKIP reduction, whereas RKIP transfection could block the Snail induction (Figure 4D). These results strongly suggest that RKIP is first and most-upstream regulator in silica-induced signaling cascade. Indeed, MPM cell lines showed the elevated expression of Snail (Figure 4E). Moreover, elimination of Snail using siRNA [25] could induce p53 expression in H28 (Figure 4F).

So, we tested the favorable effect of GN25, the small chemical inhibitor of Snail-p53 binding, on MPM cell lines. Treatment of GN25 could induce p53 more obviously in H28 (Figure 4G and 4H), comparing to positive control cell line A549. However, H2452 did not show the induction of p53 in response to GN25 (Figure 4G). Consistently with p53 induction, cell viability was also decreased by GN25 in H28 and A549 (Figure 4I). Although we observed partial reduction of cell viability in H2452 by GN25, it seems to be not related with p53 (Figure 4I). Indeed, GN25 can induce cell death partially via p73, family protein of p53 (unpublished data).

### NF2 is mediator between Snail and RKIP

**Figure 5:**
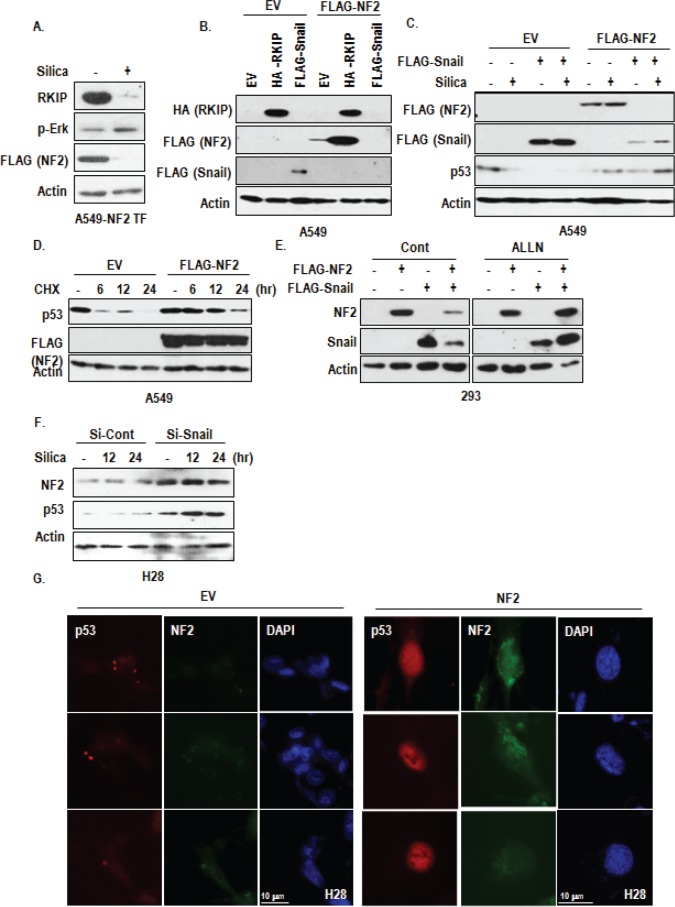
Involvement of NF2 in RKIP-Snail-p53 network (A) NF2 expression is reduced by silica. When RKIP was reduced by silica (10 μg/ml, 6 hr), NF2 expression was also drop-downed. (B) RKIP transfection induces NF2 expression. FLAG-NF2 was co-transfected with RKIP and Snail into A549 cells. Each vectors were transfected for 24 hr. (C) Snail did not co-exist with NF2 expression. In co-transfected cell with NF2 and Snail, both proteins were disappeared. FLAG-NF2 was co-transfected with Snail into A549 cells. Each vectors were transfected for 24 hr, and then silica was treated for 24 hr. And WB analysis was performed with indicated antibodies. EV indicated the empty vector, and Actin was used as loading control. (D) NF2 induces p53 expression. A549 cells were transfected with FLAG-NF2 for 24 hr and incubated with cyclohexamide (CHX; 100 μg/ml) for 6 to 24 hr. WB analysis was performed. Actin was used as loading control. (E) Proteasome inhibitor blocks NF2-Snail reduction in co-transfected cells. 293 cells were transfected with Snail and/or NF2 for 3 hr and incubated with 6 hr with 100 μM of ALLN. (F) Snail knock down induces NF2 expression in H28. At the same sample of Figure 4f, we checked the expression of NF2. (G) NF2 transfection increases nuclear p53 expression in H28 cell line. FLAG-NF2 was transfected for 24 hr. The cells were stained with anti-p53 (DO-1; red), NF2 (green), DAPI (blue).

Although we revealed that reduction of RKIP promoted Snail-mediated p53 suppression, relevance between RKIP and Snail has not been clearly demonstrated. So, we checked the interaction between RKIP and Snail. However, we did not observe the interaction (Supplementary Figure S4A). So we searched additional factor that could link RKIP and Snail and tested the involvement of NF2 that is also known to be frequently mutated in MPM and related with MAPK signaling and migration [26-28]. In A549, silica could suppressed the expression of transfected NF2 as strongly as RKIP (Figure 5A). In addition, NF2 in H28 was rapidly degraded by proteasome (Supplementary Figure S4B). In fact, RKIP could induce NF2 expression, whereas Snail suppress it (Figure 5B). We also observed the elimination of Snail when NF2 was co-transfected (Figure 5B). To confirm this, we co-transfected NF2 and Snail in A549 and 293 cells and found that NF2 as well as Snail were obviously reduced when they were co-transfected (Figure 5C and Supplementary Figure S4C). To get more detail evidence about reduction of Snail and NF2, we transfected Snail as dose dependently into NF2 transfected cells and found that NF2 expression was gradually decreased following expression of Snail (Supplementary Figure S4D). Reversely, dose-dependent decrease of Snail could be detected by increase of NF2 transfection (Supplementary Figure S4E). In addition, p53 reduction (Figure 5C) and p-Erk induction by silica and Snail (Supplementary Figure S4C) were abolished by NF2 transfection. These results indicate that reduction of NF2 is important for silica/snail-mediated Erk activation and p53 suppression. In fact, NF2 could induce p53 expression (Figure 5D). Considering that proteasome inhibitor (ALLN) could block the reduction of Snail and NF2 (Figure 5E), low expression of NF2 in MPM (in particular, H28) would be resulted from Snail expression. To test this, we checked the expression of NF2 in si-Snail transfected H28 and found that consistently with p53 induction, NF2 was increased by Si-Snail (Figure 5F). Moreover, transfection of NF2 into H28 could induce p53 expression in nucleus (Figure 5G). These results indicate that, in MPM, low expression of NF2, which would be resulted from RKIP reduction, would be reason for p53 inactivation and snail elevation.

### NF2 blocks Snail-p53 interaction

**Figure 6:**
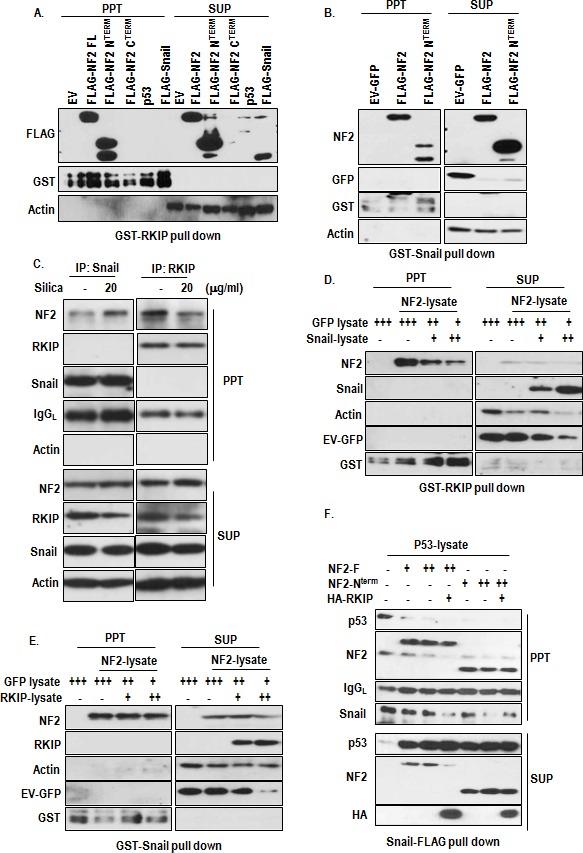
The role of NF2 in RKIP-Snail-p53 network (A) NF2, in particular, N-terminal domain interacts with RKIP. Agarose-conjugated GST-RKIP was incubated with NF2, p53 or Snail transfected HEK293 lysate in RIPA buffer for 1 hr. PPT indicated co-precipitated materials with bead-conjugated RKIP, whereas SUP indicated the supernatant. (B) Snail interacts with NF2 through N-terminal domain. The interaction was checked by GST-pull down assay. (C) IP analysis for determining of RKIP-NF2 and Snail-NF2 binding. A549 cells were incubated with silica for 2 hr and subjected into IP analysis with Snail Ab (left panel) or RKIP Ab (right panel). To prevent reduction of NF2 and Snail, cells were incubated with proteasome inhibitor, ALLN for 4 hr. (D) Snail disrupt the interaction of RKIP and NF2. At the same condition with above, increase of Snail lysate could block the interaction of NF2 and RKIP. (E) RKIP cannot disrupt the interaction between Snail and NF2. Under the same GST-pull down condition with above, increasing the RKIP-lysates did not block the interaction of Snail and NF2 (F) NF2 blocks the interaction of Snail and p53. The indicated protein was incubated with whole-cell extracts from A549 cells transfected with the indicated vectors in RIPA buffer for 1 hr. PPT indicated proteins that co-precipitated with bead-conjugated RKIP, whereas SUP indicated the supernatant. Proteins were co-precipitated by anti-Snail Ab. IgG was used for immunoprecipitation control.

To know the working mechanism of NF2 in RKIP and Snail linkage, we performed the GST-pull down assay and found that RKIP could associate with NF2 through N-terminal region (Figure 6A) and Snail was also interacted with NF2 (Figure 6B). However, Snail-RKIP interaction was not detected in this assay, consistently with previous result (Supplementary Figure S4A). To confirm the interaction, we performed the IP analysis. Consistently with our GST-pull down results, interaction between NF2 and Snail was increased by silica-treatment, whereas RKIP-NF2 binding was reduced (Figure 6C). Since RKIP and Snail were commonly associated with N-terminal region of NF2, we tested the competition between them. Increase of Snail could reduce the interaction of NF2 and RKIP (Figure 6D), whereas RKIP did not diminish the binding of Snail and NF2 (Figure 6E), indicating that Snail-NF2 binding is stronger than RKIP-NF2 and increase of Snail by additional signaling such as TGF-β or MAPK activation [29, 30] might reduce RKIP-NF2 binding, resulted in destabilization of NF2. In contrast, increase of NF2 could sequester the Snail and recover the p53 from Snail-mediated inactivation. In fact, NF2 (full length as well as N-terminal region) could block the interaction of Snail and p53 (Figure 6F).

## DISCUSSION

Human malignant pleural mesothelioma (MPM) is known to be induced by inhalation of asbestos. However, until now, clear molecular carcinogenesis model has not been established. Our main concern is how human MPM is evoked. Since asbestos cannot be supplied by commercial company, we tested the biological effect of silica. In fact, despite so many kinds of genetic studies, oncogenic mutations, responding to MPM cell proliferation, has not been proposed. Since asbestos or silica, once inhaled, are not eradicated, we assumed that existence of these materials itself would be one of tumorigenic force and may replace genetic mutation such as p53 or pRb. In fact, treatment of silica could induce p-Erk (Figure 1B) that is one of character of MPM [31]. As the mechanism for p-Erk increase, we observed the RKIP reduction (Figure 2). Although RKIP is well known inhibitor of Raf kinase, it can also block the MEK1/2 activation [32]. In fact, silica-induced p-Erk was not suppressed by MEK1/2 inhibitors (Figure 1E). Thus, RKIP is one of very plausible target. Moreover, RKIP reduction by silica seemed to be very general event, even in non-cancer cell lines (Figure 2C). Although, until now, we do not know clear mechanism about RKIP reduction by silica, continuous existence of silica can suppress RKIP expression and may drive transformation to MPM.

In previous report, RKIP has been suggested as important target gene for p53-induced senescence [23]. In fact, RKIP can promote cellular senescence in response to genotoxic stress-p53 pathway. However, in this study, we found that RKIP could work as upstream activator of p53. Indeed, overexpression of RKIP could increase p53 expression in A549 (Figure 5B). However, this result should be carefully interpreted because A549 possesses oncogenic K-Ras and basically, p53 is suppressed by Snail [25]. In fact, RKIP-mediated p53 induction is achieved by inhibition of Snail by RKIP-NF2 network and not general event in other kinds of cells.

Concerning low mutation of p53 in MPM, this study provides basic clues. In H28, p53 is inhibited by Snail. In fact, p53 was restored by si-snail (Figure 4F), RKIP transfection (Figure 3F), treatment of GN25 (Figure 4G and 4H) or NF2 overexpression (Figure 5G). Since H28 is classified as sarcomatoid (10-20% of MPM) [33, 34], that is very aggressive and less responding to chemical treatment, GN25 would be one of drug candidate for H28 like MPM. Although we also observed the reduction of cell viability in H2452 by GN25 (Figure 4I), it would be resulted from p53 independent mechanism. In fact, GN25 can activate p73-mediated apoptosis (unpublished data). Thus, we next checked the involvement of p73 in GN25-induced cell death in H2452.

**Figure 7:**
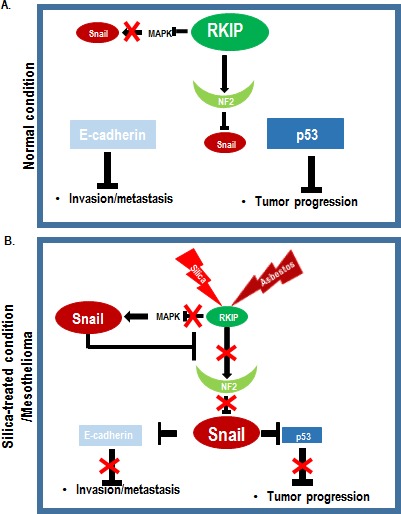
Summarized Diagram (A) Under normal condition, RKIP block the Snail through MAPK inhibition and NF2 stabilization. Thus, p53 and E-cadherin exert tumor suppressive functions. (B) When silica or asbestos are inhaled, they suppress RKIP and sequentially reduce NF2 stability. Inactivation of RKIP-NF2 network might induce Snail-mediated p53 suppression as well as E-cadherin down-regulation. Thus, long-term exposure to silica or asbestos will evoke mesothelioma.

In this study, we also revealed the novel function of NF2, inhibitor of snail. In fact, NF2 is one frequently mutated genes in MPM. However, its molecular role has not been clearly demonstrated. NF2 is linked various cell signaling including cell-cell junction and Hippo pathway [35]. Indeed, we could observe the increase of cell size by NF2 transfection (Figure 5G) that is one of important feature of Hippo pathway [35]. However, in this study, NF2 was cellular inhibitor of Snail, in particular, Snail-mediated p53 suppression. Under normal condition, NF2 may inhibit Snail to prevent cell migration. This speculation is very plausible because NF2 is tightly linked to cell-cell adhesion and works as migration inhibitor [28, 35]. If RKIP is reduced by silica or other reasons, NF2 would be reduced by destabilization. In contrast, under the RKIP induced condition (such as p53 activation), NF2 may be stabilized and inhibit Snail-mediated p53 and E-cadherin suppression (Figure 7). In MPM model, RKIP reduction or inactivation by cytosolic aggregation (Figure 2F) and rapid digestion in lysosome (Supplementary Figure 2E and F) may weaken NF2 function. In addition, if Snail is elevated by several triggers (For example; activation of TGF-β signaling [29, 30], RKIP reduction in MPM or K-Ras activation in pancreatic cancer [25]), the interaction of RKIP and NF2 (Figure 6E) would be disrupted and p53 is suppressed (Figure 7).

Our results are consistent with previous hypothesis that cytotoxic stresses (in our case, silica or asbestos) provide selective pressure and resistant clones against this pressure by genetic or non-genetic signaling alternation (RKIP reduced or NF2 mutated cells) would be founder of cancer [36]. In fact, treatment of silica in A549 obviously suppresses cell proliferation. However, reduction of RKIP provides resistance to silica and enable to clonal selection. So, at early stage of MPM, many cells would be eliminated by silica-induced toxicity. However, small clones that express low RKIP will be selected and expanded to MPM.

In summary, in human MPM, silica or asbestos that is retained in lung may provide the tumor-promoting ability through reduction of RKIP (Figure 7). Since NF2 expression is dependent on RKIP, RKIP reduction may promote NF2 inactivation, sequentially induce Snail. Activated Snail will bind and inhibit p53 and also block the RKIP-NF2 binding. Long-term exposure of silica/asbestos, finally, induce invasive cancer because of E-cadherin reduction. Thus, Snail-p53 binding inhibitor such as GN25 or Snail inhibitor would be useful for treatment of MPM.

## MATERIALS AND METHODS

### Cell lines and reagents

Silica (S5631; silicon dioxide) was purchased from Sigma Aldrich (St, Louis, Mo, USA). ALLN, MG132, U0126 were provided by Calbiochem (Darmstadt, Hessen, Germany). PD98059 was purchased from Stressgen (San Diego, CA, USA). GN-25 was provided by Dr. Song GY (Chungnam National University) [37]. A549, AGS, H1299, H28, H2452 and HEK293 cell lines were obtained from American Type culture collection (ATCC, Manassas, VA, USA) and maintained in RPMI-1640 or DMEM containing 10% FBS and antibiotics. NSCLC cell lines (NCI-H23, NCI-H322, NCI-H358, NCI-H460) and lung fibroblast cell lines (WI-26, L132) were kindly provided by Dr. Kim S (Seoul National University). SCLC cell lines (NCI-H69, NCI-H146, NCI-H209) were purchased from Korean Cell line Bank (KCLB, Seoul, Korea) and maintained in RPMI-1640 containing 10% FBS and antibiotics. HCT116 and its isogenic p53−/− cell lines were obtained from Dr. Vogelstein B (Johns Hopkins University) and maintained in RPMI-1640 containing 10% FBS and antibiotics.

### Western blot analysis

Protein was extracted from cells in RIPA buffer (150 mM NaCl, 25 mM Tris-Cl, 1% NP-40, 1% sodium deoxycholate, 0.1% Sodium dodecyl sulfate (SDS), containing protease inhibitor cocktail). After sample buffer was added to lysates, the mixtures were heated at 95°C for 7 min. Samples were applied to Sodium dodecyl sulfate-polyacrylamide gel electrophoresis (SDS-PAGE) and western blot (WB) analysis was performed according to general protocol. The following antibodies were used in this study: HA (sc-7392), His (sc-8036), GFP (sc-9996), GST (sc-138), RKIP (sc-5426), SNAIL (sc-28199), p53 (sc-126), NF2 (sc-331), β-catenin (sc-7963), Actin (se-1616) and E-cadherin (sc-8426) were purchased from Santa Cruz biotechnology (Santa Cruz, CA, USA). Anti-HER2/Neu (MAB3782) and anti-FLAG (F3165) was obtained from Millipore (Billerica, MA, USA) and Sigma Aldrich (St, Louis, Mo, USA), respectively.

### Immunofluorescence staining

Cells grown on coated cover glasses were fixed with 100% MeOH for 1 hr at −20°C. After washing with Phosphate bufferd saline (PBS) and blocking in PBS based buffer (containing normal human IgG (1: 200; 30 min at 4°C)) to eliminate non-specific reaction, cells were incubated with primary antibodies (1: 100~1: 200; overnight at 4°C) and sequentially with suitable either FITC (Fluorescein isothiocyanate) or Rhodamine-conjugated secondary antibodies for 6 hr at room temperature. DNA was stained with 4, 6-diamidino-2-phenylindole (DAPI). Endoplasmic reticulum was stained by ER-TrackerTM dye (Invitrogen, California, USA, MP12353). After washing with PBS, cover glasses were mounted with mounting solution (Vector Laboratories, Burlingame, CA, USA; H-5501). Immunofluorescence (IF) signal was detected through fluorescence microscope (Zeizz, Jena, Germany).

### Recombinant proteins, immunoprecipitation and GST pull-down assays

For the analysis of protein-protein interaction, Glutathione S-transferase (GST)-pull down assay and Immunoprecipitation (IP) assay were performed. For GST-pull down, agarose-bead conjugated GST (negative control) or GST-target protein was incubated with cell lysate or recombinant protein in RIPA buffer for 1 h at 4°C. IP assay was performed with cell lysate or recombinant protein with RIPA buffer. The whole lysates were incubated with proper first antibodies for 2 hr at 4°C and reacted with agarose bead conjugated protein A/G (Invitrogen, Carlsbad, CA, USA) for 2 h. After centrifugation, precipitated materials were washed with RIPA buffer twice and subjected into SDS-PAGE and WB analysis.

### MTT assay

To determine the cell viability, MTT (3-(4, 5-Dimethylthiazol-2-yl)-2,5-diphenyltetrazolium bromide) assay was performed. Cells were incubated with 0.5 mg/ml of MTT solution (Calbiochem) for 4 hr at 37 ^o^C. After removing the excess solution, the precipitated materials were dissolved in DMSO (Dimethyl sulfoxide) and quantified by measuring the absorbance at 540 nm. For cell proliferation, cells were seeded in 12well-plate and were counted by hematocytometer. Cell proliferation was determined by two independent experiments.

### Transfection of vectors and si-RNAs

pCMV-RKIP-HA was provided by Keum G (David Geffen School of Medicine at University of California, Los Angeles, CA, USA). Snail vector were provided by Dr. Hung MC (MD Anderson cancer center, TX, USA The pcDNA3 NF2-FLAG (Isoform 1 of merlin) [38], pcDNA3 NF2-N^TERM^-FLAG 1-332 AA, pcDNA3 NF2-C^TERM^-FLAG 308-595 AA were obtained from Addgene (Cambridge, MA, USA). HA-p53 vectors were kindly gift from Dr. Kim S (Seoul National University, Seoul, KOREA). For *in vitro* gene knock down, si-RNA against target proteins were generated (Cosmo Genetech, Seoul, Korea). Target sequences of si-RNA for each gene are described at Table1. For transfections, we used the jetPEI transfection agent (Polyplus Transfection, New York, NY) following the manufacturer's protocol. The vector (1.5 μg) was mixed with 1.5 μl of jetPEI reagent in 150 mM NaCl solution. After incubation for 15 minutes at room temperature, the mixture was added to the cell. After 3 hr, the serum-free medium was replaced with 10% FBS–containing medium.

### RNA isolation and RT-PCR

For reverse transcription polymerase chain reaction (RT-PCR), total cellular RNA was extracted using RNA extraction kit (Qiagen, Maryland, USA). After measurement of RNA concentration, 1 μg of total RNA was reverse transcribed to cDNA using MMLV RT (Invitrogen, California, USA) and random hexamer. RT-PCR was performed with specific primers of target genes. The sequence of primers used in this study are available upon request.

## SUPPLEMENTARY FIGURES


