# Supplementary material for: NF2 blocks Snail-mediated p53 suppression in mesothelioma
Source: Oncotarget. 2015 Mar 12;6(12):10073–85. doi: 10.18632/oncotarget.3543 (PMC4496341; doi:10.18632/oncotarget.3543)
Supplement: Supplementary file 1 [file oncotarget-06-10073-s001.pdf]

NF2 blocks Snail-mediated p53 suppression in mesothelioma

Supplementary Figure legends

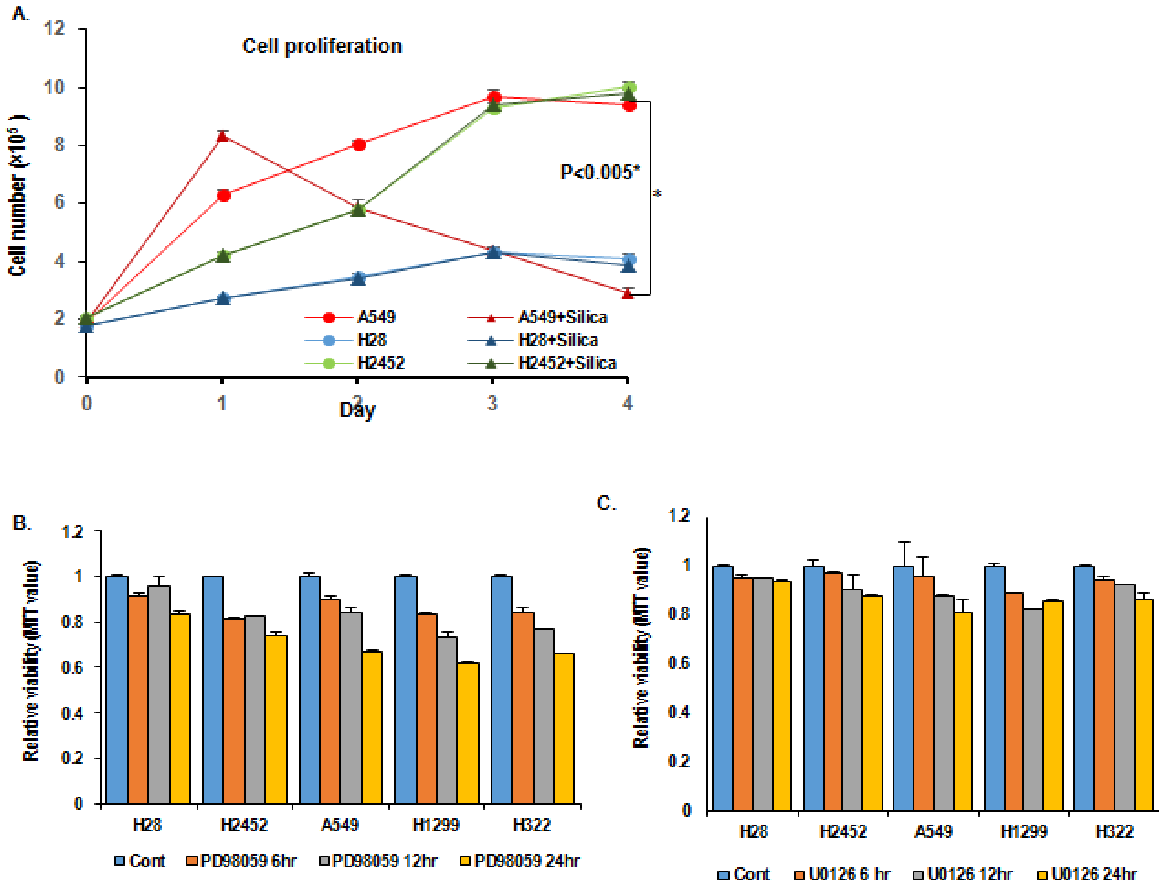

Supplementary Figure S1: (A) The different response in cell proliferation between MPM cell lines and A549. Each cell line was seeded at $2x10^5$ cells/well and cultured with or without 10 µg/ml of silica for 4 days. Every 24hr, cells were counted through hemocytometer. Graph showed the average cell number from 3 independent experiments. **(B and C)** MAPK inhibitors cannot suppress MPM viability. PD98059 (5 µg/ml) and U0126 (2 µM) were treated by the hour. The cell viability was monitored by MTT assay.

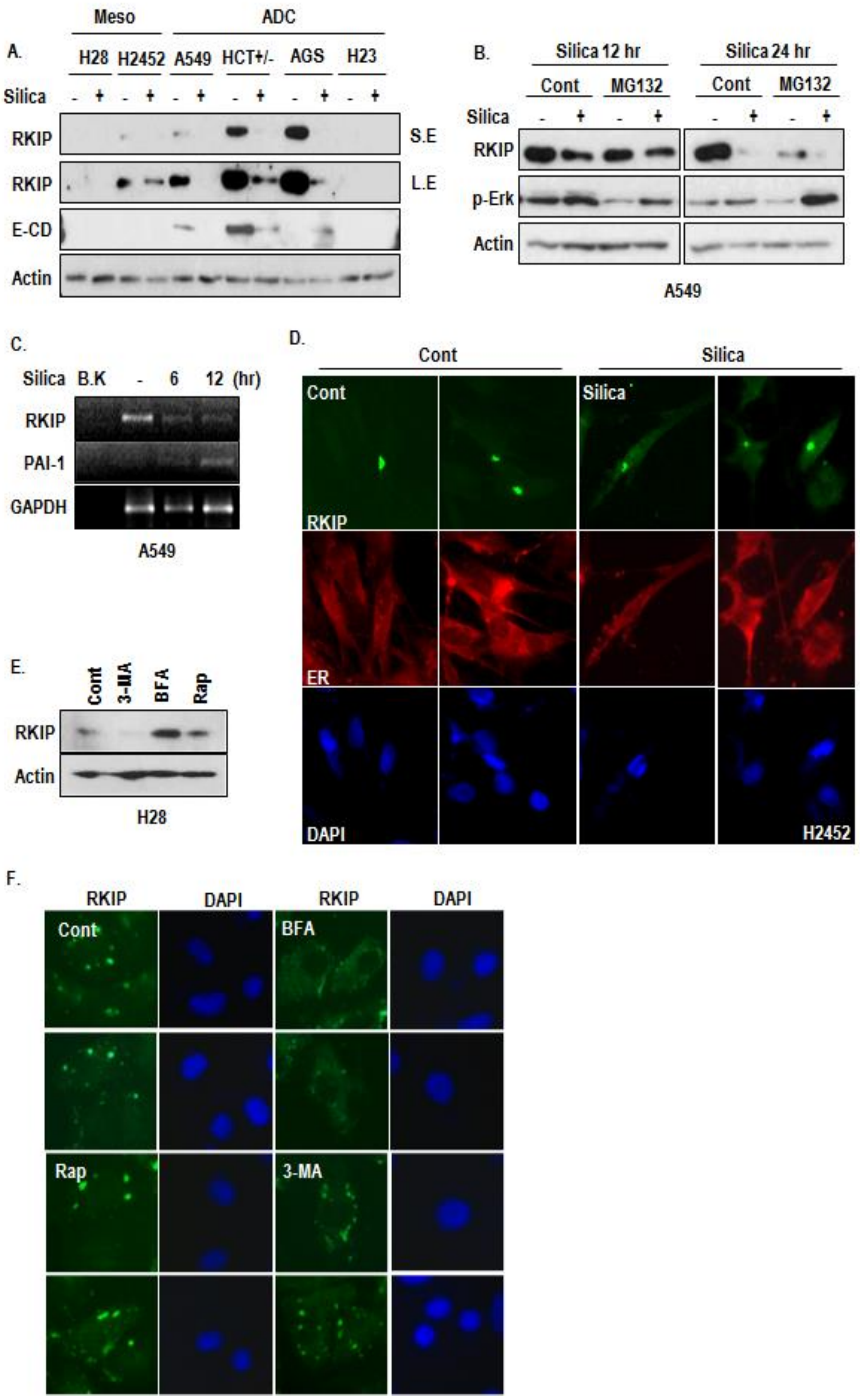

Supplementary Figure S2: (A) RKIP expression is obviously suppressed in adenocarcinoma cell lines (HCT116, AGS, and H23). Silica was treated for 24 hr in SF condition. WB analysis was performed with indicated antibodies and Actin was used as loading control. S.E and L.E indicated short and long-term exposure, respectively. **(B)** Proteasome inhibitor does not block the RKIP

reduction by silica. A549 cells were treated with proteasome inhibitor (MG132) before an hour to be treated silica. Reduction of RKIP was monitored by WB analysis treatment for indicated time. Actin was used as loading control. **(C)** PAI-1 transcript is increased by silica. Induction of PAI-1 was monitored by RT-PCR after treatment for indicated time in A549. RT-PCR was performed to confirm each transcript expression using matched specific primers. BK indicates blank reaction (without cDNA), and GAPDH was used as loading control. **(D)** RKIP forms small spots in cytoplasm of H2452. H2452 cells were stained with RKIP (green), DAPI (blue) and ER (red). (**E**) Lysosome but not autophagy inhibitor can induce RKIP expression in H28. To know the reason of low RKIP expression, H28 was incubated with 3-MA (autophagy inhibitor; 5 mM), Bafilomycin A1 (BFA; lysosome inhibitor; 5 μg/ml) and Rapamycin (Rap; mTOR inhibitor; 50 μg/ml) for 2 hr. Among them, BFA could induce RKIP expression. (**F**) BFA blocks cytoplasmic spotting of RKIP. At the same condition, RKIP was diffused in cytosol by BFA, but not by Rap and 3-MA.

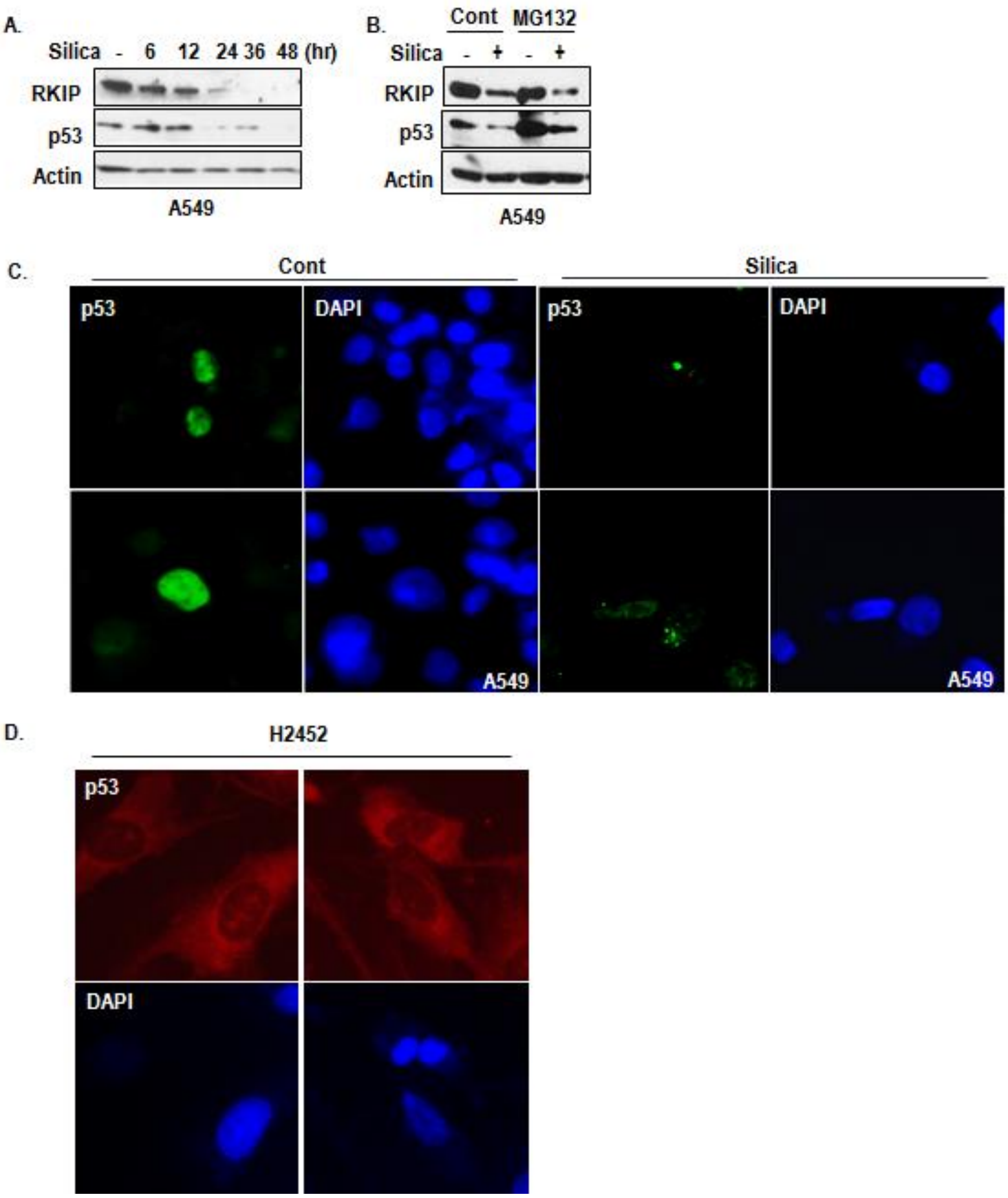

Supplementary Figure S3: (A) p53 shows the similar expression kinetics with RKIP in response to silica in A549. Reduction of p53 and RKIP was monitored by WB analysis after treatment for indicated time in A549. **(B)** RKIP regulates p53 expression as proteasome-independent mechanism. A549 cells were treated with proteasome inhibitor (MG132) before an hour to be treated silica. Silica was treated for 24 hr in SF condition. WB analysis was performed with indicated antibodies, and Actin was used as loading control. **(C)** p53, in

response to silica, is detected in cytoplasmic as a small vesicle. A549 cells were treated with silica for 24 hr in SF condition and stained anti-p53 (DO-1; green), DAPI (blue). **(D)** p53 in H2452 is diffused in cytoplasm. The cells were stained with anti-p53 (DO-1; red), DAPI (blue).

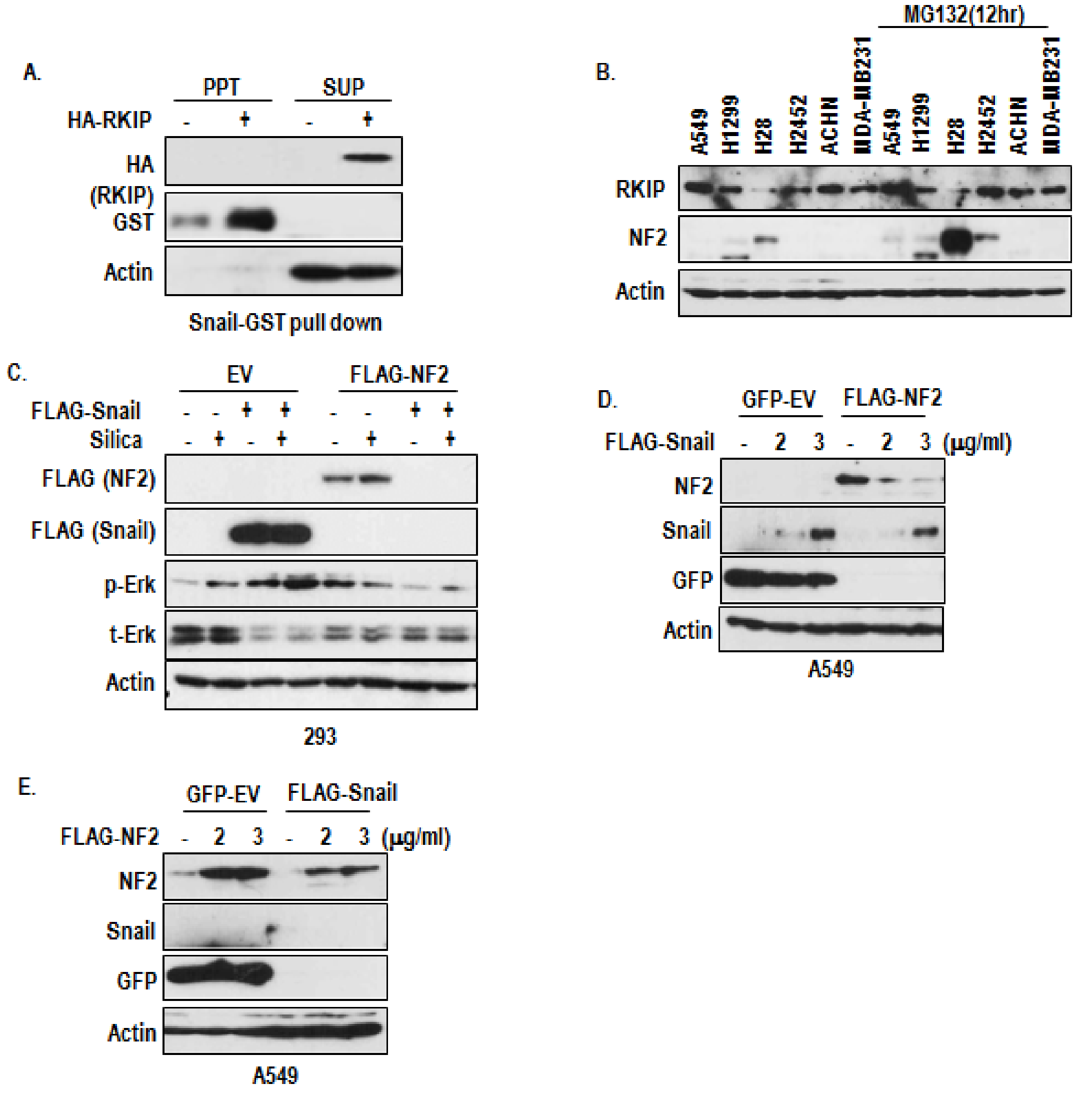

Supplementary Figure S4: **(A)** RKIP and Snail do not show binding affinity. Using GST-Snail-bead and RKIP-transfected cell lysate, GST pull-down assay was performed. There was no co-precipitated RKIP with Snail-bead (PPT), whereas RKIP was detected in supernatant (SUP). **(B)** NF2 expression is increased by proteasome inhibitor in H28 and H2452. Each cells were treated with proteasome inhibitor (MG132) for 12 hr, and WB analysis was performed with indicated antibodies. **(C)** Snail did not co-exist with NF2 expression. In co-transfected cell with NF2 and Snail, both proteins were disappeared. FLAG-NF2 was co-transfected with Snail into 293 cells. Each vector was transfected for 24 hr, and then silica

was treated for 24 hr. And WB analysis was performed with indicated antibodies. EV indicated the empty vector, and Actin was used as loading control. **(D)** Snail can suppress NF2 expression as dosage-dependent manner. GFP or NF2 expressed 293 cells were transfected with increasing dose of Snail for 24 hr. following increase of Snail, NF2 expression was declined. GFP was used for transfection control and did not show obvious reduction by increase of Snail expression. **(E)** Increase of NF2 can suppress Snail expression. In this case, we could not also reduction of GFP by increase of NF2.
